# Severe Tetanus Following a Rooster-Peck Injury Requiring Nasotracheal Intubation: A Case Report

**DOI:** 10.5811/cpcem.53949

**Published:** 2026-06-29

**Authors:** Robin White, Hardeep Singh, Kelsey Craver, Rebecca Cranston, Kelsey Hodge, Elizabeth Vandervort

**Affiliations:** * Northeast Georgia Medical Center, Gainesville, Georgia; † Northeast Georgia Medical Center, Graduate Medical Research, Gainesville, Georgia; ‡ Northeast Georgia Medical Center, Department of Emergency Medicine, Gainesville, Georgia

**Keywords:** tetanus, trismus, airway management, immunization, case report

## Abstract

**Introduction:**

Tetanus is a rare but life-threatening disease caused by *Clostridium tetani*, characterized by generalized muscle rigidity, autonomic instability, and respiratory compromise.

**Case Report:**

We present a case of a 65-year-old male poultry hobbyist with type two diabetes who developed severe tetanus following a rooster-peck injury to his finger. The patient presented with progressive trismus and respiratory distress necessitating fiberoptic nasotracheal intubation and subsequent tracheostomy. Despite appropriate antimicrobial therapy, immunoglobulin administration, and sedation, the patient continued to exhibit spasms when sedation was reduced.

**Conclusion:**

This case highlights the ongoing risk of tetanus in under-immunized agricultural populations, the challenges of airway management in severe trismus, and the importance of maintaining vaccination awareness even in developed healthcare settings.

## INTRODUCTION

Tetanus is caused by the neurotoxin *Clostridium tetani*, which blocks inhibitory neurotransmission, leading to muscle rigidity and spasms.^1^ Despite the availability of vaccination, sporadic cases continue to occur in unvaccinated or inadequately immunized adults, particularly after minor contaminated wounds.^2^ In the United States, most cases are seen in older individuals and those with incomplete immunization.^3^

Animal-related injuries are rarely implicated in tetanus transmission, yet agricultural workers are at higher risk due to increased chronic environmental exposure to spores via sources such as soil and manure.^4^ This case underscores the importance of early recognition, vaccination, and multidisciplinary management in a patient with severe tetanus following a seemingly trivial poultry injury.

## CASE REPORT

A 65-year-old male poultry hobbyist with a history of type two diabetes mellitus and no tetanus vaccination within the prior 10 years presented to the emergency department (ED) with trismus, abnormal tongue sensation, and dysarthria. He reported a left third-finger injury from a rooster peck several days prior, which had developed signs of cellulitis (Image). He denied dyspnea or difficulty handling secretions. Physical examination revealed trismus, hypertension, tachycardia, and a small erythematous finger wound.

Computed tomography (CT) of the brain and CT angiography of the head and neck were negative for stroke, deep-space infection, peritonsillar abscess, retropharyngeal abscess, or cellulitis of the neck, while patient history lowered suspicion for a dystonic reaction to home medications and serum calcium ruled out hypocalcemic tetany. He was treated with diphenhydramine for its anticholinergic effects in consideration of less likely but possible dystonic reaction without improvement. Additionally, the patient was given diazepam for spasms and trismus, and trimethoprim-sulfamethoxazole for his finger wound. He was then discharged home with return precautions. As per chart review, vaccination status did not appear to have been known on this initial presentation to the ED.

The following morning, the patient re-presented via emergency medical services with worsening trismus, an apneic episode at home witnessed by family, chest pain, and perioral paresthesias. On arrival, he was in acute respiratory distress with oxygen saturation dropping to 35% on room air, tachycardia, tachypnea, and labile blood pressures. The patient’s venous blood gas lactate level (normal: 0.00 – 1.90) was elevated to 9.5 millimoles per liter (mmol/L) from 2.3 mmol/L the prior day. Despite benzodiazepines, trismus persisted, and the patient exhibited difficulty clearing oral secretions. His family was unaware of his last tetanus vaccination, but presumed it was longer than 10 years prior.

Given the risk of hypoxia and difficult airway, anesthesia and trauma teams performed fiberoptic nasotracheal intubation, which was successfully placed after initial left mainstem intubation. Considering exclusion of likely differentials for tetanus during the patient’s prior ED visit, he promptly received a tetanus booster and metronidazole, vancomycin, piperacillin-tazobactam for both *Clostridium tetani* coverage and broad-spectrum coverage of cellulitis by animal bite. He was then admitted to the intensive care unit (ICU) with a diagnosis of severe generalized tetanus and administered tetanus immune globulin 1,500 units.

On day two, a plastic surgeon was consulted for evaluation of the rooster peck wound and need for debridement for toxin source control. Wound culture was performed and revealed *Escherichia coli*, *Staphylococcus simulans*, and *Staphylococcus saprophyticus*. An infectious diseases consultant was also called on arrival at the ICU, contributing to management and narrowing antibiotics to only ceftriaxone and metronidazole by day three.

*CPC-EM Capsule*

What do we already know about this clinical entity?

*Tetanus follows contaminated wounds and can cause trismus, spasms, and respiratory failure; risk rises with poor vaccination and diabetes.*

What makes this presentation of disease reportable?

*Tetanus after a rooster peck is rare; poultry injuries are seldom reported sources of* Clostridium tetani *infection.*

What is the major learning point?

*Even minor animal-related injuries can cause severe tetanus; always assess vaccination status and provide prophylaxis.*

How might this improve emergency medicine practice?

*This case promotes early recognition, vaccination review, and timely tetanus immune globulin in atypical wounds to prevent intensive care unit-level complications.*

On day four, the patient underwent surgical tracheostomy by the trauma surgery team for long-term airway management. The ICU therapy included ongoing monitoring by the infectious diseases consultant and continuous sedation with propofol, fentanyl, midazolam, and ketamine, along with methocarbamol, magnesium sulfate, and diazepam for muscle spasm control. Attempts to wean sedation resulted in recurrent spasms and autonomic instability. He also required norepinephrine infusion for sedation-related hypotension.

As of the writing of this case report, the patient remained in the ICU receiving supportive care and gradual weaning from sedation.

## DISCUSSION

This case illustrates several clinically significant points regarding the recognition and management of tetanus in medical practice. While tetanus most commonly follows contaminated puncture wounds, animal-related injuries are rarely reported. Poultry pecks are an uncommon vector but may inoculate *Clostridium tetani* spores present in soil-contaminated beaks or claws. The patient’s type 2 diabetes may have contributed to impaired wound healing and increased susceptibility to infection, underscoring the importance of vaccination and early wound care, especially in diabetic patients exposed to agricultural environments.^5^

In the United States, high serologic immunity (~94%) indicates generally strong protection against tetanus, although levels are lower among adults ≥ 80 years of age.^6^ National surveys also show racial and ethnic disparities in both primary vaccination with tetanus, diphtheria, and acellular pertussis (Tdap) and booster vaccination with tetanus and diphtheria (Td) with Black and Hispanic adults reporting lower adult Tdap coverage compared with White adults.^7^ In Georgia, middle-aged adults (50–64 years of age) report higher Td/Tdap coverage than both younger adults (18–49 years of age) and older adults (> 65), according to the state vaccine advisory committee profile.^8^ Additionally, sex differences are evident with women having higher adult vaccination rates than men, in part because Tdap is commonly administered during pregnancy.^9^

Tetanus is primarily a clinical diagnosis. Its early presentation of trismus and dysarthria mimic other neuromuscular and infectious conditions, which combined with the rarity of tetanus disease in the United States (only 187 reported cases from 2016 to 2022) and our patient’s unknown vaccination status, likely contributed to the delay in diagnosis and treatment.^10^ This emphasizes the importance of maintaining high clinical suspicion for tetanus in unvaccinated, under-vaccinated individuals or individuals with unknown vaccination history presenting with trismus or dysphagia, with or without overt wounds, especially in the context of diabetic patients who are at greater risk for infection and complications.^11^

Although orotracheal intubation is significantly more common, fiberoptic nasotracheal intubation remains an effective, reasonable technique for airway management in severe tetanus with profound trismus limiting mouth opening and minimizes risk of laryngospasm and trauma to the airway.^12^ Early tracheostomy, as performed in this case, is recommended as it allows for better airway control and secretion management during the prolonged ICU stay often required for patients with severe generalized tetanus.^13^

Intensive care unit management involves multidisciplinary approaches and focuses on controlling spasms (benzodiazepines, propofol, magnesium, muscle relaxants), eradicating toxin production (metronidazole), and neutralizing circulating toxin (tetanus immune globulin).^14^ Vasopressor support may be needed due to heavy sedation and autonomic instability. Despite optimal care, recovery is often prolonged, with persistent spasm risk for several weeks.^15^

This case report is limited by its single-patient nature and lack of confirmatory testing for *Clostridium tetani* toxin. Management decisions, therefore, were based on clinical diagnosis and multidisciplinary judgment. Additionally, the patient’s ongoing ICU course precluded full assessment of long-term neurologic and functional recovery.

## CONCLUSION

This case underscores the importance of continued clinical vigilance for tetanus in under-vaccinated adults presenting with trismus and dysarthria, particularly those with risk factors such as diabetes mellitus or consistent exposure to soil or animals. Even minor wounds can serve as a portal of entry for *Clostridium tetani*. Additionally, this case highlights the need for immunization awareness, prompt recognition of tetanus disease, rapid administration of immunoglobulin, early airway planning, and aggressive supportive care with multidisciplinary ICU management to optimize outcomes in severe tetanus.

## Figures and Tables

**Image.**
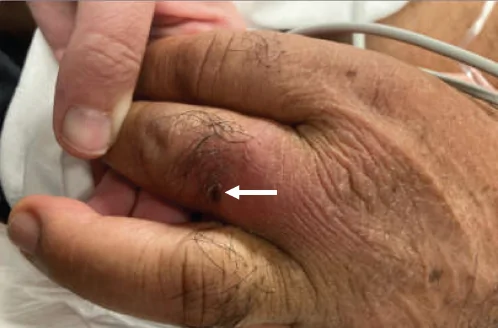
Rooster-peck injury of left third finger with early signs of cellulitis present (erythema, warmth, tenderness, swelling). Arrow indicates site of rooster peck.
